# Gender and sociocultural factors in animal source foods (ASFs) access and consumption in lower-income households in urban informal settings of Nairobi, Kenya

**DOI:** 10.1186/s41043-022-00307-9

**Published:** 2022-07-11

**Authors:** Salome A. Bukachi, Mariah Ngutu, Ann W. Muthiru, Aurélia Lépine, Suneetha Kadiyala, Paula Domínguez-Salas

**Affiliations:** 1 Institute of Anthropology, Gender and African Studies (IAGAS), University of Nairobi (UoN), IAS Museum Hill, Nairobi, Kenya; 2 University College London (UCL), Gower Street, London, WC1E 6BT UK; 3 London School of Hygiene and Tropical Medicine (LSHTM) & Leverhulme Centre for Integrative Research On Agriculture and Health (LCIRAH), Keppel St, Bloomsbury, London, WC1E 7HT UK; 4 Natural Resources Institute (NRI), University of Greenwich, Medway Campus, Central Avenue, Chatham Maritime, Kent, ME4 4TB UK; 5 International Livestock Research Institute (ILRI), Naivasha Rd, Nairobi, Kenya

**Keywords:** Gender, Sociocultural, Social norms, Decision-making, Animal source foods (ASFs)

## Abstract

**Background:**

Gender shapes household decision-making and access for nutritious diets, including animal source foods (ASFs) that impact on child health and nutrition status. However, research shows that the poorest households in the urban informal settlements of Nairobi have low ASFs consumption. This study was conducted to explore further from a qualitative perspective the gender, sociocultural factors affecting household ASF consumption this study.

**Methods:**

To explore further on the topic of study, an exploratory qualitative study was carried out to establish the factors that influence access, allocation and consumption of animal source foods (ASFs) by households in urban informal settings of Nairobi. Nineteen focus group discussions with men and women were conducted to enable in-depth exploration of ASFs consumption.

**Results:**

Gender influences decision-making of household ASFs dietary intake. Gendered power dynamics prevail with men as breadwinners and household heads often determining the food access and consumption of ASFs. Women are increasingly accessing short-term waged-based incomes in urban informal settings and now play a role in food and nutrition security for their households. This enforces the idea that women’s decision-making autonomy is an important aspect of women empowerment, as it relates to women’s dietary diversity and subsequently, better household nutritional status. As evidenced in this study, if a woman has bargaining power based on accessing incomes to support their household food needs, she will not jeopardize food security. The mobile digital money platform was key in enabling access to resources to access food. Use of trust to access food on credit and purchasing smaller packaged quantities of food were also enablers to access of food/ASFs.

## Background

Food insecurity is still prevalent in many parts of the world with gender disparities identified as one of the key drivers especially in developing countries [1]. Despite ongoing efforts to alleviate food insecurity in Kenya, estimates indicate that approximately 50% of Kenyans are food insecure with 10% in constant need of food relief [2]. A key component of food insecurity is the lack of access to a sufficient quantity of nutritious food especially animal source foods (ASFs) which is a potential risk factor for malnutrition in children and adults [1]. Animal source foods are high-quality nutrient-dense products that supply essential amino acids, vitamins and minerals, to prevent micronutrient deficiencies including stunting and anemia and promote cognitive development [3, 4]. Food consumption is highly regulated by social, cultural, environmental and economic factors, and ASFs tend to be among the most regulated across all settings [1, 5–7]. In most settings where differential consumption patterns do occur, the differences tend to be on luxury foods (non-staple foods), such as ASF, rather than necessity foods (staple foods) [4, 8]. Previous research showed that the lower-income households in Nairobi’s urban informal settlements had low ASFs consumption [4, 9].

There is evidence to support the impact of ASFs on the nutrition and health status of marginal populations including those in informal settings around the world [1, 2, 4, 10]. Harris-Fry et al. point out that ASFs even in relatively small quantities can make a significant difference in the nutrition and health of children and adults; increased ASFs intake is linked to many different types of health outcomes; and ASFs are an important component of dietary quality and diversity, even when economic factors are controlled [6]. Various studies on ASFs consumption have shown that access to these food groups within the household or community depends on age, gender and power relations, which, in turn, affect the ability of an individual’s nutrition situation [1, 5, 9–12].

However, there is limited evidence on how gender interplays with factors of ASFs access, allocation and consumption in households and their effect on nutrition. Control over cash income, decision-making and access to ASFs are all part of the gender issues that are shaped and intertwined with, and within the broader socio-ecological system that defines men and women in society.

With a surge in urban informal settlements, there are overcrowding and resultant challenging environments that lack accessible roads, health, housing water and sanitation services. Availability of and access to safe nutritious food are a major challenge for populations in these lower-income settings [4]. Using dietary diversity among households in lower-income urban informal settings, Dominguez-Salas et al. found that socioeconomic and gender factors are critical when addressing the question of food security from the demand side [10]. Other factors hypothesized in the literature to negatively influence food security include family size and dependency ratio [2, 4, 6, 10]. This places the population, especially women of reproductive age and young children, at a risk of micronutrients deficiencies such as anemia and stunting [1, 2, 4].

Food security has been a dominant focus in development interventions and research, with the gendered dimensions receiving increasing attention [4, 13, 14]. Ultimately, the dynamics between gender and associated factors including power, social systems, and complex food landscapes influences decision-making on the dietary intake to dictate how much of what kind of food including ASFs is consumed when and by whom in the households. Food, including ASF, access allocation and consumption patterns in households indicate specialized knowledge of specific members and the responsibilities and roles attached to certain subjectivities. This paper presents findings of an exploratory study that examined gender and sociocultural factors affecting household ASF access and consumption in informal settlements in Nairobi.

## Methods

### Study setting and sample population

The study was conducted in Dagoretti sub-counties (North and South) which are peri-urban, with low-income residential settlements in Nairobi city County, Kenya’s largest capital. The study sites have lower population density than other areas of Nairobi, but with a higher density of livestock. This has over time led to the multiplicity of ASF value chains and hence diversity, a key consideration for the study setting selection. The study participants were purposively selected and consisted of couples (men and women of reproductive age) recruited from households with children between 6 and 60 months of age in the informal settlements of Dagoretti North and South, Nairobi County.

### Data collection

We utilized qualitative focus group discussions (FGDs) to inform the exploratory study design. Through the FGDs, the study examined in-depth the gender, social norms and cultural factors that affect household ASF consumption in low-income resource-limited settings. A total of nineteen focus group discussions (7 groups of men and 12 groups of women) each constituted of 12 discussants.

The FGD guide used for the discussions was designed based on the Agriculture for Nutrition and Health (ANH) Food Environment framework (FEWG) [12] focused on the personal food environment domain. These were reflected in the structure, style and content of the focus groups discussions which were framed with open-ended questions relating to dietary practices and household decision-making around food including access, allocation and consumption of ASFs. Targeted probing questions based on participants’ responses allowed for a participant-directed discussion.

All the focus group discussions were conducted by trained research assistants working in pairs as moderator and note taker, respectively. Swahili, the national language, was the medium of communication in the focus group discussions given that the participants represented multiplicity of ethnic backgrounds with varying vernacular languages but were well conversant in Swahili.

### Ethical considerations

The research assistants took the study participants through the informed consent form and the information sheet provided to all participants before the start of the discussions. Questions and concerns from the participants were addressed before written consent was obtained. Besides handwritten notes, the FGDs were also recorded using digital audio recorders with permission from the study participants to help capture the discussions.

Names of discussants and places that were identifiers were replaced with pseudonyms on the transcripts for anonymity and confidentiality of the study participants.

### Data analysis

The study utilized the gender and intersectionality approach to guide the analysis hinged on the inductive approach in grounded theory. Audio recordings from the FGDs were later reviewed and compared to the field notes captured by the note taker to ensure data quality. Data were transcribed alongside the field notes and translated into English transcripts for coding and analysis. The transcripts were reviewed for quality. A coding framework was developed following a review of the transcripts to identify emerging codes. Coding was done on NVivo software, and themes were directly drawn from the data to inform the study questions.

## Results

### Gender roles and responsibilities in access to ASFs

In terms of accessing ASFs, there were different possibilities across the households interviewed and these were often defined by traditional, social and cultural definitions of roles and responsibilities of men and women across ethnicities and backgrounds represented in the informal settings.

In most discussions, men talked of their role as providers of money to meet the family’s needs, while the women’s role included decisions on what food to purchase and prepare. Most men did not want to get involved in the kitchen work as food preparation is a task traditionally assigned to women. Men also seemed to have a general feeling that women are responsible for buying vegetables, eggs, milk, cheaper meat varieties like the chicken pieces [*kata kata*] and small fish [*omena]* often costing from as low as KES 20, while the men are responsible for purchasing for home use, meat from the butchery that is often of higher value. This could suggest that men had complete control of when to consume specific ASFs particularly meat. This is supported further when male discussants talked about buying meat as they returned home from work, sending money to their wives or leaving them with some money in the morning for purchasing meat later in the day.

Men indicated giving their wives money for food to enable them plan a daily meal schedule to fit within the resources. In the majority of the household’s women managed the food budget: *‘In my house, … he can provide a specific amount of money then it is me who will budget using that cash… it is me who decides what to do with it and how long it will last.’ (Women’s FGD 12).* Both men and women shared the perspective of men as the designated providers and women as the managers of food in their households including ASFs: ‘*For me it is my wife who decides [what to purchase] while I provide the money.’ (Men’s* FGD06).

In a few households, men kept the money and made purchases of food items, including ASFs on a daily or weekly basis: ‘*If its vegetables and tomatoes, it’s the woman who is supposed to purchase them but if its meat then the man is the one to purchase. The woman can be sold meat with a lot of bones [because she is not an expert in choosing choice cuts of meat] but the man knows the good places to get good/choice meat.’ (*Men’s FGD01). This was especially common where the women were stay-at-home mothers with no source of income. In such households, the distribution of bargaining power between spouses was unequal as the ability of women to make choices was often constrained by their spouses’ preferences and value as sole breadwinners: *You know sometimes we can look at it from two different perspectives. One where I am the one who goes to look for income and sometimes I give my wife money to go and purchase food or sometimes I ask for the list of what is needed and purchase the food including meat* (Men’s FGD03).

### Gender and social norms in ASFs allocation and consumption

The ASF access, allocation and consumption practices were often fueled by cultural beliefs and social norms, which were often similar across Kenya’s numerous ethnic communities and persist even in urban settings. Using chicken as an example, participants referred to it to illustrate dimensions of gender and culture in food allocation and consumption: ‘*Let me speak only on chicken. If we are children in a house, we are not supposed to eat “imondo” [Gizzard]. The “imondo” is preserved for the head of the family while the children eat the legs and the “matumbo” [offals].’ (Men’s FGD08*). Allocation of specific choice parts of the ASFs was preserved for men, as women and children were either prohibited from eating some choice parts of the ASFs as illustrated: *‘We were brought up believing that the chicken gizzard was tasty and therefore it was for men and not women.’ (Women’s FGD07*).

On the intra-household allocation of ASFs, women from the culturally diverse FGDs were of the view that men came first in terms of order of serving, size of portions and parts of ASFs to be consumed. It was evident that serving men first was perceived as a sign of respect to the spouse’s position as the head of the household.

*‘[In terms of serving food], I will start with my spouse because he is the head of the house and then the children*. *The best way is for me to serve the children [to avoid embarrassments], for example if I have cooked chicken or meat, the child will be quick to serve what is meant for the man. That's why it is good for the mother to serve everyone.*’ (Women’s FGD 13). Women and children seemed to have similar consumption habits which may disadvantage their consumption of ASFs. The perception of men as breadwinners was occasionally given as one of the reasons for being served first and with the largest ASF entitlements in the household. Men were also of similar perspectives that they are ordinarily prioritized in ASF preference and allocation in the households: ‘*… you will find when it comes to chicken being placed on the table, there are those parts that the father is supposed to eat. So he will be served first then the rest can follow’* (Men’s FGD07). They also emphasized the role of women in the allocation of food: ‘*It is mostly the woman of the house who serves food. Circumstances where men serve themselves are rare* (Men’s FGD 01).

### Changing gender and social norms in ASFs access and consumption

Socioculturally driven perceptions define men as the main providers for household budgets including food, but women’s contribution was necessary to supplement the men’s income to meet the household needs including food. While it was culturally not supported, some men were increasingly acknowledging and embracing the contribution of women to household budgets including purchase of food in the constrained resource settings: *‘The way the economy is currently, the wife should also have a part to play in a contribution. If you contribute three hundred, she should also contribute fifty shillings.’* (Men’s FGD 08)

*‘The house is for both of you, the wife and the spouse. There is no third person, so you need to help each other. Marriage is about helping each other and not relying on one person.’* (Men’s FGD 07).

Through the participation of women in household food budgets, consumption of ASFs in the urban informal settings would increase: *‘We are eating more ASFs because we do not sit and wait [for our husbands to purchase food]. If I got a casual job and got two hundred shillings, I would buy the ASFs instead of waiting for my spouse to bring it.’* (Women’s FGD 10). Ordinarily, men dependent on wage-based incomes would leave some money behind for their wives to purchase food. However, in situations where they did not have money, they would go look for casual jobs and immediately they got paid; they would utilize mobile money transfers to send their wives the money. This would apply to those households where both the man and woman own a phone. With mobile money transfers, it is possible to meet family food needs by sending money so that it is available at the needed time: ‘*I can say this, buying food is not a problem it depends on the environment, where you are…. You can be far from your wife, but you can send her money through mpesa to purchase food….’ (Men’s FGD02).* Use of the mobile money services has helped avert such situations where ‘*You can come home with meat you arrive at 10.00 pm when the children are asleep; will they benefit? You see, they will just hear that in our house there was meat ….’* (Men’s FGD 19). While mobile money transfers were helpful, there were instances where households did not have enough money but wished to consume ASFs. At such times, a good relationship for both the men and women with a retailer would come in handy to enable access to ASFs like milk and meat on credit: ‘*I will buy from one retailer because I know if I don’t have cash I can purchase my goods on credit.’* (Women’s FGD09)

Buying smaller portions of ASFs was a way of coping with limited financial resources for several households. Women would, for instance, purchase meat/beef for as little as KES 50 to supplement their meals: *‘These days we have devices that measure the kilos and give the prices. So, we can buy according to how much money one has. For example, … you can afford fifty shillings’ worth of meat and you cook with Sukuma[kales] and share with your family.’* (Women’s FGD 11)

Opting for cheaper varieties of ASFs was also one of the ways households coped with limited income. In the case of chicken, men would buy small pieces of chicken such as, feet, necks, gizzards, and this would in their case satisfy the craving to consume chicken. When these were bought it did not matter what part of a chicken it was, it counted just as chicken altogether and men would feel fulfilled regardless as children would be happy to eat ‘chicken’ even if just as pieces, and the men would derive their satisfaction from being able to provide*:* ‘*Children also like kata kata and they are cheap. For instance, if you buy chicken feet worth fifty shillings, and it is cooked well, children will be satisfied and feel like they have eaten chicken*.’ (Male FGD 07)

*‘So that the children don’t complain that they never eat chicken. They say, “Dad has brought us chicken.” … at that time, you have only bought about 30 chicken feet. Every child gets about 5 chicken feet. With that your children do not feel like they have been left out when maybe others [neighbouring children] have eaten chicken.’* (Men’s FGD 09)

Women too were happy to access these cheaper varieties of ASFs to meet a need for consuming a particular ASF: *‘Kata kata is good because the person who does not own chicken and is not able to purchase a full chicken, can buy chicken pieces for 30 or 20 shillings and add to the kales and they will feel like they have eaten chicken.’* (Women’s FGD 05). The cheaper ASFs varieties satisfied the preference for ASFs for families in informal settlements who are often challenged by limited incomes.

The study setting comprised mainly of short-term income opportunities for men and women in the informal sectors often requiring unskilled workers. The participants represented a population where men worked mainly as security guards, construction casual laborers, public service vehicle drivers and touts as well as roadside vendors’ dependent on daily/weekly wages. Women found work as domestic workers on a daily wage, small business workers/owners in food kiosks, hairdressing and roadside vegetable and houseware sells. The informal and short-term nature of work opportunities and the little pay were often not enough for men to cater for the family needs, including food budgets in a setting where the population is mostly dependent on market supply*: ASFs are good and I can be including goat meat in my budget everyday but the pocket cannot allow it. So, when I am lucky [to get extra wages] I will tell my family, ‘now go and buy meat.’* (Men’s FGD 03).

*‘Even those who are married still have a problem of access to food because if that man gets money you find that it is not enough. So, the wife is forced to go hassle and get casual jobs to get additional money*’ (Women FGD06). Women reported that because of the low income, they would often need to also make their own contributions to supplement those family budgets. In this setting, women reported accessing small incomes as they doubled up as caregivers of the home and children as exemplified in the following excerpts:

*‘As a woman, you can buy food because maybe your spouse doesn’t have money and you have done some casual jobs or someone has given you something small, you will just buy food because you cannot wait for your spouse to come back, only to say he doesn’t have money.’ (Women’s FGD04)*

*‘A woman should plan for herself. If she gets some money from washing for people clothes she should keep it. If her husband comes back home and does not have any money or if he fails to come back home, the wife can plan for the children with the money she earned.’ (Women’s FGD05)*

Women alongside their spouses would find work opportunities to earn some income. These incomes were accessible through income generation activities like doing laundry or beadwork, or running small businesses like selling vegetables or second-hand clothes and at times being in women self-help groups. These odd jobs or wage labor acted as a buffer for women and their households to access food in case their spouses were not able to provide

*‘Maybe you have washed clothes for someone and have gotten some two hundred shillings, you will just have to buy[food]. You won’t wait to be told go and buy this, you just decide on your own.’ (Women’s FGD 01)*

*‘You cannot depend on your spouse. Your spouse can be having another family somewhere and you are sitting waiting for money and there is none. The children are the ones who will suffer. So, the woman should plan for herself.’ (Women’s FGD 12)*

While everyday food purchases were thought to be within the woman’s sphere, some women explained that there would be conflict or discomfort of some sort if they(women) bought meat because their spouses may not know the source of money*: ‘if she buys meat, it can lead to conflicts. The question would be, “where did you get the money to buy meat”? your wife should tell you where that luck [opportunity for income enough to buy meat] has come from.’* (Men’s FGD 07)

*‘… if a woman buys ASFs like meat, and the spouse does not know where the money came from, he cannot eat that meat. Even if you cook, he'd rather sleep hungry, or you cook for him vegetables. He believes his money is what should buy meat in the home.’* (Women’s FGD 013)

## Discussion

Within households in the study setting, the decision on what is consumed by who involved the co-evolution of practices within the fabric of social power structures reflecting intra-household power dynamics. These power positions in households played out along the axes of age and gender as social-cultural expectations performed through food including ASFs practices [3, 5, 9, 10, 15]. As illustrated in this study and in existing literature, household decision-making in relation to the purchase, allocation and consumption of ASF was mainly fueled by socioeconomic status (SES) but also informed by the intersecting gender and age considerations that drive intra-household access, allocation and consumption [3, 5, 6, 10].

Notably, whilst men were the socioculturally designated breadwinners expected to provide for the family budgets, women were increasingly supplementing household food needs through wages from casual jobs. Women in urban informal settings increasingly play a central role in food and nutrition security for their households through short-term income generation wage-based and self-organization activities. This re-enforces the idea that women’s engagement in the productive spheres contributes to their decision-making autonomy and is an important aspect of women’s economic empowerment, as it relates to women’s dietary diversity and subsequently, better nutritional status. As evidenced in this study, access to income increased women’s bargaining power and decision-making in food decisions including the purchase, allocation and consumption of ASFs. In contrast, women in households where men were the main income earners and had the overall decision-making power on ASFs purchase had limited ability to make choices on purchase of ASFs due to the fear of conflicts within the household. Similar findings were found in Timor-Leste where women were free to make everyday food purchases except ASFs where they often required their spouse’s permission before purchasing. In this study, getting ASF without consent from the men often attracted anger and, in some instances, resulted in gender-based violence [5]. Nevertheless, women in our study reported more consumption of ASFs in households where they contributed to food budgets. This implies that women’s economic empowerment can contribute to increased ASFs and be associated with household and child nutrition [16].

There is evidence that women’s participation in household decision-making and ability to purchase food including ASFs (an aspect of empowerment) is correlated with the availability of diverse diets in the household. Various elements of women empowerment and disempowerment for that matter have been linked to lower or higher nutritional risks respectively [1, 4, 10]. A study in Nepal concludes that higher SES is associated significantly with more frequent consumption of most food groups, including ASFs, in-season fruits and vegetables [9]. Also, a positive association between increases in women’s empowerment and improved nutrition outcomes has been documented and any actions leading to women’s disempowerment can result in adverse nutritional impacts for women and children limiting their access to ASFs that provide important micronutrients for nutrition and well-being. Studies in Kenya, Ghana, Bangladesh and Vietnam observed a positive association between maternal education and maternal dietary diversity [1, 4, 10, 17].

Enablers of access to ASFs in the face of limited resources included credit, opting for cheaper options or smaller packaged purchases. Often with the limited incomes in households in low-income settings and the high cost of most ASFs, cheaper lower-quality ASFs varieties were options for meeting nutrition needs. Cost has also been reported in other studies as a barrier to access of ASFs in low-income households [9, 15]. Cheaper ASFs options easily accessed in informal markets were often unvalidated for quality and safety and ultimately challenging to overall health and nutrition status in these households [6].

In situations where one did not have cash but managed to get some causal job, mobile money platforms were used to send money back home to enable immediate access to food. The availability of mobile money transfers (M-pesa) made it possible for men to better support their household food needs as emergent in the study. Indeed, the digital money platforms have been instrumental in enabling real-time access to the food through remittances [18]. Further humanitarian agencies have increasingly relied on cash transfers through these digital money platforms, and results indicate that these mobile transfers are better options for enabling agency in food access [19]. Notably, the leveraging on mutual trust between retailers and consumers to enable access to ASFs on credit matches well with the quantitative component of this study to be shortly published by the authors.

However, in this study men were seen to have strong preferences for ASFs and influenced the purchase, allocation and consumption in households. This articulates the role of gender in ASFs consumption by different members of the households. It points to the culturally influenced practice of prioritizing men perhaps to the disadvantage of women and children in the consumption of ASFs in resource-limited settings where these foods are not frequently accessed, yet are important for their well-being. The finding is similar to a study done in Uganda and Zambia which revealed that women had more control and decision-making power when it came to decisions on what to eat in relation to vegetables, whereas the men controlled decisions about consumption of animal source foods such as meat, fish and eggs [17]. This could suggest that the challenges of malnutrition can be reduced if men would also be targeted for improved nutrition programs and interventions. Nutrition programs need to include men to ensure the availability of diverse diets in the households.

Decision-making to purchase ASFs in the lower-income urban informal study setting had a larger household component than previously considered in the more high-income settings [3]. In lower-income urban informal settings, families had to consider household size and composition in terms of gender and age, factors often influenced by sociocultural norms, to determine if, when and what ASF is to be bought and consumed. Also, the preference of especially the men as head of household and the prescription of ASFs for young children also influenced the choice and consumption. This takes place in a context where the economic status already is a key factor that influences consumption. The most crucial decisional point was affordability in terms of costs, followed by food preferences of spouse and children. On intra-household allocation and consumption of ASFs, differences in the allocation and consumption of food especially based on age and gender were seen in this study as also captured in other contexts as defined by social-cultural norms. Age-based differences in consumption of ASFs do exist, but the form differs based on sociocultural backgrounds represented in the study setting. In food allocation, men were considered more than women and children. This is consistent with the findings of a study done in Uganda and Zambia that found that the allocation of more ASFs to men was often defined by how a wife was expected to behave as well as social perceptions on men’s hard work and their role as providers [20].

Cultural norms, taboos and beliefs lie within the contextual factors included as some of the key basic causes of malnutrition [21, 22]. Local beliefs and cultural practices on ASFs choice and consumption as illustrated in this study prioritized men and limited particularly ASFs consumption by women and children [7]. A study done in Uganda showed that providing men with larger portions of meat was seen as an encouragement for men to continue buying meat for the household. Women were also afraid that if they did not follow the custom, their spouses might leave them and go to women who gave them more meat [20]. Studies in western societies also report similar finding between gender and specific foods, where meat, especially red meat, alcohol, and large portion sizes are associated with masculinity, while vegetables, fruit, fish and sour dairy products are associated with femininity [23, 24]. Looking further into the prescriptions and proscriptions of ASF especially for women and children would inform the design of sociocultural and gender-sensitive programs and interventions targeted at tackling malnutrition in such settings.

Ultimately as detailed in this study, gender, age and sociocultural factors are seen to intersect with attitudes and practices to influence ASFs dietary intake for households in lower-income urban informal settings [5]. It is also undeniable that economic status including the contribution of women to household budgets at the household level is tied to access to ASFs. This essentially affects how much women and children access and benefit from ASFs-based diets to address malnutrition.

## Limitations

There are two limitations in this study. First, there are several ASFs available in the study area, but this study focused only on chicken because this was the ASFs that drew a lot of discussion when it came to gender, social norms and culture. Future studies can explore the gendered aspects of other ASFs. Second, the cross-sectional exploratory design of this study means that causal relationship between gender and factors associated with it could not be established. Future studies are needed to understand and confirm the associations in this study. Nevertheless, the study findings provide useful qualitative information on gender and ASFs access, allocation and consumption in households in informal settlements from which future studies can draw from and build upon.

## Conclusions

Gender and intra-household decision-making intersect with household economic status and sociocultural practices to drive ASFs (and food more broadly) access for women and children. Gendered relations mediate processes of decision-making on the access, allocation and consumption of ASFs, and these could further affect the nutritional status of different members within the households. Men are the main contributors to household food budgets and key in purchase of ASFs. However, women also play a role albeit minimal in supplementing the household food budget by engaging in odd jobs to raise some money. Gender and sociocultural norms influence ASFs access, allocation and consumption. Gender roles and responsibilities at the household pit men at the center of purchase of ASFs and women at the center of their preparation. Men are the key decision-makers in the purchase of ASFs; however, when it comes to purchase of small quantities of ASFs whose costs are low, women also participate in their purchase. Culture and social norms in relation to ASFs access, access, allocation and consumption are gendered though slowly changing given that the current economic times hence have implications on individual dietary intake especially for women and children. Through these results and discussions, this paper contributes to relevant research on associations between gender and other factors and how these influence ASFs dietary intake for households in lower-income urban informal settings. It also has implications for nutrition-sensitive programs seeking to address the underlying causes of undernutrition in women and children in low-income resource-limited settings.

## Recommendations

Further research to include rural–urban settings is necessary to inform the association of gender and other factors and how these influence ASFs dietary intake for households on a broader more diverse scale. Given the importance of gender and social norms in consumption of ASFs, the design of nutrition programs including education modules need to incorporate gender and social–cultural intersecting factors to inform access, allocation and consumption of ASFs to help address the underlying causes of malnutrition (undernutrition) in women and children in low-income resource-limited settings.

Another key consideration is ensuring that during the design of nutrition programs, a thorough gender analysis is carried out before implementation to understand the context and current situation of a community in relation to gender and nutrition to enable development of appropriate local solutions.

Women were seen to help support the household access to food and ASFs. Strengthening women’s efforts in supporting household nutrition through women empowerment programs such as microfinance programs, rotating savings and credits schemes and training on income generating activities would go a long way to increase household access especially for women and children to ASFs.

Men are key in household access to ASFs, hence the importance of their inclusion as important actors in household nutrition programs. Some of the strategies would involve engaging male champions in the community to serve as role models and disseminators of messages on the importance of active participation of men in nutritional programs. This would help in debunking deeply rooted beliefs on the consumption of animal source foods by different household members.

The use of the digital mobile money platforms to send money in the informal settlements provides an opportunity that can be explored for its use in nutrition education, awareness creation and sensitization of men and women in the community.

## Data Availability

The datasets used and/or analyzed during the current study are available from the corresponding author on reasonable request.

## Abbreviations

ANH: Agriculture for Nutrition and Health
ASFs: Animal source foods
FGDs: Focus Group Discussions
FAO: Food and Agriculture Organization of the United Nations
FEWG: Food Environment framework
SES: Socioeconomic status
IAGAS: Institute of Anthropology, Gender and African Studies
LSHTM: London School of Hygiene and Tropical Medicine
LCIRAH: Leverhulme Centre for Integrative Research on Agriculture and Health
NRI: Natural Resources Institute
UoN: University of Nairobi
ILRI: International Livestock Research Institute
